# Effects of combined radiofrequency radiation exposure on levels of reactive oxygen species in neuronal cells

**DOI:** 10.1093/jrr/rrt116

**Published:** 2013-10-08

**Authors:** Kyoung Ah Kang, Hyung Chul Lee, Je-Jung Lee, Mi-Na Hong, Myung-Jin Park, Yun-Sil Lee, Hyung-Do Choi, Nam Kim, Young-Gyu Ko, Jae-Seon Lee

**Affiliations:** 1 Research Center for Radio-senescence, and Division of Radiation Cancer Research, Korea Institute of Radiological and Medical Sciences, Seoul 139-706, Korea; 2 College of Pharmacy and Division of Life Science and Pharmaceuticals, Ewha Womans University, Seoul 120-808, Republic of Korea; 3 EM Environment Research Team, Electronics and Telecommunications Research Institute, Daejeon 305-700, Korea; 4 School of Electrical and Computer Engineering, Chungbuk National University, Cheongju 361-763, Korea; 5 School of Life Sciences and Biotechnology, Korea University, Seoul 136-713, Korea

**Keywords:** combined RF radiation, menadione, H_2_O_2_, reactive oxygen species, neuronal cells

## Abstract

The objective of this study was to investigate the effects of the combined RF radiation (837 MHz CDMA plus 1950 MHz WCDMA) signal on levels of intracellular reactive oxygen species (ROS) in neuronal cells. Exposure of the combined RF signal was conducted at specific absorption rate values of 2 W/kg of CDMA plus 2 W/kg of WCDMA for 2 h. Co-exposure to combined RF radiation with either H_2_O_2_ or menadione was also performed. The experimental exposure groups were incubator control, sham-exposed, combined RF radiation-exposed with or without either H_2_O_2_ or menadione groups. The intracellular ROS level was measured by flow cytometry using the fluorescent probe dichlorofluorescein diacetate. Intracellular ROS levels were not consistently affected by combined RF radiation exposure alone in a time-dependent manner in U87, PC12 or SH-SY5Y cells. In neuronal cells exposed to combined RF radiation with either H_2_O_2_ or menadione, intracellular ROS levels showed no statically significant alteration compared with exposure to menadione or H_2_O_2_ alone. These findings indicate that neither combined RF radiation alone nor combined RF radiation with menadione or H_2_O_2_ influences the intracellular ROS level in neuronal cells such as U87, PC12 or SH-SY5Y.

## INTRODUCTION

As a large number of individuals have been exposed to the radiofrequency (RF) signals from cellular phones, social concerns regarding the possible biological effects of RF radiation emitted from cellular phones on human health have been increasing. While a large part of the world, including Europe, Australia and East Asia, use wideband code division multiple access (WCDMA), several countries use the code division multiple access (CDMA), and some countries, such as Korea, use both [1]. However, information about the biological effects of combined RF radiation exposure has not been well established. Due to humans often being simultaneously exposed to multi-signal RF radiation, we have recently focused our research on the possible biological effects of exposure to multi-signal RF radiation [1–3].

Incomplete one-electron reduction of oxygen, such as singlet oxygen, superoxides, peroxides, hydroxyl radical and hypochlorous acid, form the reactive oxygen species (ROS). ROS causes oxidative modification of DNA, proteins, lipids and small intracellular molecules, which are associated with cell or tissue damage, and are the contributing factors for cellular events including gene expression, cell proliferation, apoptosis, differentiation and senescence [4]. Furthermore, overproduction of ROS plays an important role in several diseases, including cancer, diabetes, cardiovascular diseases, aging and neurodegenerative diseases such as Alzheimer's and Parkinson's diseases under both physiologic and pathologic conditions [5, 6]. Therefore, balancing of the intracellular ROS level plays an important role in preventing thes pathophysiological conditions.

A number of studies have been made into possible adverse biological effects of RF fields on oxidative stress. While most studies have shown no effects of RF radiation on ROS formation [7–11], some studies have recently reported that RF radiation emitted from mobile phones could promote ROS production in some cell types including neuronal cell models. Due to the proximity of mobile phones to the head and the electrical activity of the brain, the nervous system may be a preferential target for the study of RF radiation effects on biological substrates. Several studies have explored the effects of exposure on nervous systems including the SH-SY5Y neuroblastoma and SN56 cholinergic cell lines, as well as rat primary cortical neurons [12–20]. Enhancement of chemically induced ROS production in neuroblastoma cells has been demonstrated after 872 MHz CW RF radiation exposure, but not after GSM-modulated RF radiation exposure [18]. Höyto *et al*. (2008) [19] also reported that the GSM-modulated signal enhanced chemically induced oxidative stress, but that RF radiation alone did not enhance oxidative stress in neuroblastoma cells. Another study reported that the neurotoxic effects of hydrogen peroxide were exacerbated by RF radiation exposure in SN56 cells but not in primary cortical neurons, suggesting that the RF signal acts as a co-stressor for the oxidative damage of neural cells under particular circumstances [16]. Xu *et al*. (2009) [20] reported that RF radiation alone significantly increased ROS production, thereby leading to further oxidative damage to mitochondria DNA in primary cultured neurons.

Information related to the biological effects of multiple RF radiation is still very minimal, and studies related to the RF radiation effect on oxidative stress are still controversial. In order to further investigate this issue, we examined the possible effects of combined (837 MHz and 1950 MHz) RF radiation on the intracellular ROS level in neuronal cell culture systems. The present study focused on co-exposures, i.e. combining RF radiation with chemicals that induce oxidative stress, in U87, PC12 and SH-SY5Y cells as neuronal cell models.

## MATERIALS AND METHODS

### RF radiation exposure system

The Radial Transmission Line (RTL) exposure system was used as an *in vitro* multi-frequency radiation exposure system for this study. The details about this exposure system have been described previously [3]. A typical CDMA signal of 837 MHz and a WCDMA signal of 1950 MHz were applied to the RTL after amplification.

### RF radiation exposure protocol

For these experiments, cells were plated in plastic 65-mm cell culture dishes ∼ 16 h prior to each exposure. The cell count (2 × 10^6^) seeded per dish was selected from preliminary experiments to obtain a subconfluent culture at the end of each exposure. The exposure system was then warmed up for 2 h to equilibrate it prior to RF exposure. The petri dishes (8 dishes/exposure) were placed within the exposure chamber. Exposure of RF radiation for combined (CDMA at 2 W/kg plus WCDMA at 2 W/kg) signals was performed for 2 h. During the exposure period, the temperature in the chamber was maintained within a range of 37 ± 0.3°C by circulating water within the cavity. The temperature of the culture media was monitored twice per second throughout the exposure period in the sham- and the RF radiation-exposed groups. A temperature-controlled mixture of air and CO_2_ (5% CO_2_ inside the chambers) was provided by ventilation from a modified cell culture incubator. For the sham exposure, the cells were kept in the RF radiation exposure device, but were not exposed to RF radiation. The cells were exposed in six groups: (i) incubator control group, (ii) sham exposure group, (iii) RF radiation exposure group, (iv) ROS inducers (in the form of hydrogen peroxide (H_2_O_2_) (Sigma Chemical Co., St Louis, USA) or menadione (Sigma Chemical Co., St Louis, USA)) exposure group, (v) sham + ROS inducers co-exposure group, (vi) RF radiation + ROS inducers co-exposure group. After 2 h RF radiation exposure, the cells were immediately transferred to a cell culture incubator and further analyzed at 1, 3, 6 and 12 h after H_2_O_2_ treatment, or at 0.5, 1 and 3 h after menadione treatment.

### Cell cultures

The NIH3T3 mouse fibroblast cells, U87 human glioma cells, PC12 rat pheochromocytoma cells and SH-SY5Y human neuroblastoma cells were purchased from American Type Culture Collections (ATCC) (Manassas, VA, USA). NIH3T3 cells were maintained in Dulbecco's modified Eagle's medium (DMEM) (HyClone, Logan, UT, USA) supplemented with 10% fetal bovine serum (FBS; Invitrogen, Paisley, Scotland, UK) and 25 U/ml penicillin/streptomycin. U87 cells were cultured in DMEM supplemented with 10% FBS (WelGENE, Daegu, Korea) and 25 U/ml penicillin/streptomycin. PC12 cells were maintained in DMEM medium supplemented with 10% horse serum (Invitrogen, Paisley, Scotland, UK), 5% FBS, and 25 U/ml penicillin/streptomycin. SH-SY5Y cells were cultured in a 1:1 mixture of ATCC-formulated Eagle's Minimum Essential Medium (ATCC, Manassas, VA, USA) and Ham's-F12 medium (Invitrogen, Cergy Pontoise, France) supplemented with 15% FBS and 25 U/ml penicillin/streptomycin. Cells were kept at 37°C in a cell culture incubator with a humidified atmosphere of 5% CO_2_.

### Analysis of intracellular ROS levels

To quantify intracellular ROS production the fluorescent probe 2′7′-dichlorofluorescein-diacetate (DCFH-DA) was employed. It is a non-polar compound that easily penetrates the cell membrane and is hydrolyzed by intracellular esterases to its nonfluorescent polar derivate DCFH. In the presence of ROS, DCFH is oxidized to the fluorescent dichlorofluorescein (DCF) [21]. After exposure to either RF radiation alone or RF radiation with ROS inducers, the cells were washed twice with chilled PBS and incubated with 10 µM DCFH-DA at 37°C for 15 min in the dark, followed by washing twice with chilled PBS. Cells were trypsinized and analyzed through a Flow Cytometer (Beckton Dickinson, Franklin Lakes, NJ, USA) with excitation and emission at 490 and 530 nm respectively.

### Statistical analysis

All values were expressed as the mean value of duplicates of at least six independent experiments ± SD. The results were subjected to an analysis of the variance (ANOVA) using the Tukey *post hoc* test for the multiple comparisons (SigmaStat 3.1; Systat Software Inc., Chicago, IL, USA) in the incubator control, sham-exposed and the RF-exposed groups with or without ROS inducers. Values of *P* < 0.05 were considered as significantly different from either sham-exposed or incubator control, respectively.

## RESULTS

### Effect of combined RF radiation exposure on intracellular ROS levels in neuronal cells

**Fig. 1.**
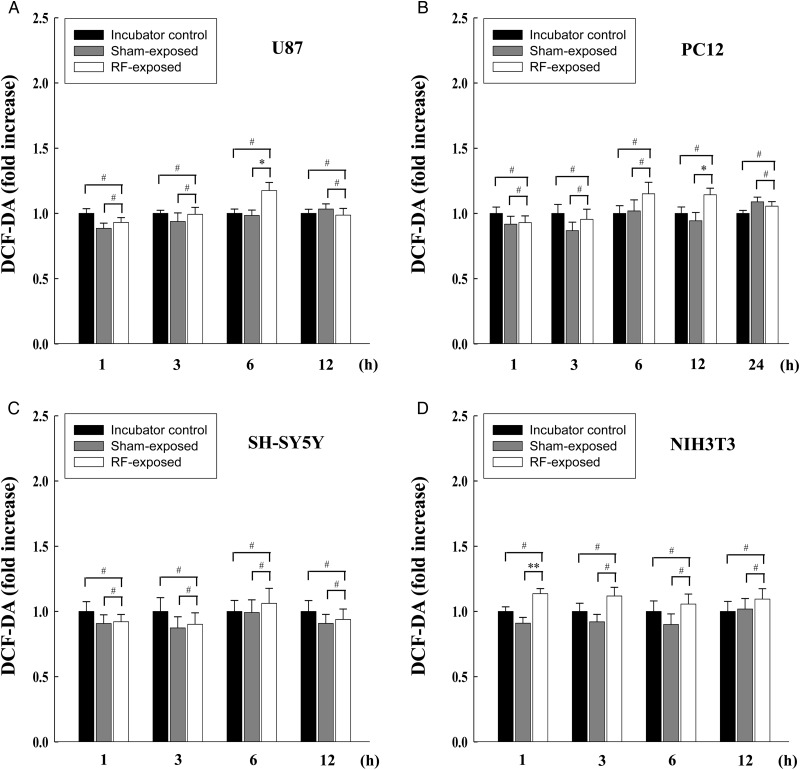
Intracellular ROS levels after exposure of neuronal cells to combined RF radiation alone (CDMA at 2 W/kg plus WCDMA at 2 W/kg for 2 h). Intracellular ROS levels were measured by DCF-DA staining in (**A**) U87, (**B**) PC12, (**C**) SH-SY5Y, and (**D**) NIH3T3 cells at 1, 3, 6 and 12 h (additional 24 h in the case of PC12 cells) after exposure. The data are expressed as the means of six independent experiments together with the standard deviations of the means (M ± SD). Statistical calculations were performed by ANOVA via the Tukey *post hoc* test. Statistical significance values were *P < 0.05 and **P < 0.01, compared with either the incubator control or sham-exposed groups. # indicates non-significance (*P* > 0.05).

To examine whether combined RF radiation (CDMA at specific absorption rate (SAR) of 2 W/kg plus WCDMA at SAR of 2 W/kg for 2 h) affects the intracellular ROS levels in neuronal cells, we measured ROS levels by DCF-DA staining in three different neuronal cell lines (U87, PC12 and SH-SY5Y) at various time-points (1, 3, 6 and 12 h) after combined RF exposure (Fig. 1). ROS levels in U87, PC12 and SH-SY5Ycells did not show any changes with the exceptions of 6 h post RF-exposure in U87 cells and 12 h post RF-exposure in PC12 cells (Fig. 1A–C). The increased ROS level at 12 h post RF-exposure was not sustained until 24 h post RF-exposure in the PC12 cells. NIH3T3 mouse embryonic fibroblasts were included in every experiment for this study as an alternative type of control cells. In NIH3T3 cells, ROS levels after combined RF exposures were unchanged except for 1 h post RF-exposure (Fig. 1D). Every ROS level was analyzed by ANOVA using the Tukey *post hoc* test for the multiple comparisons. Our results indicated that the ROS level was neither consistently nor sustainably affected by the combined RF radiation exposure, with statistical significance in four different types of cell lines.

### Effect of H_2_O_2_ on ROS levels and cell viability in neuronal cells

**Fig. 2.**
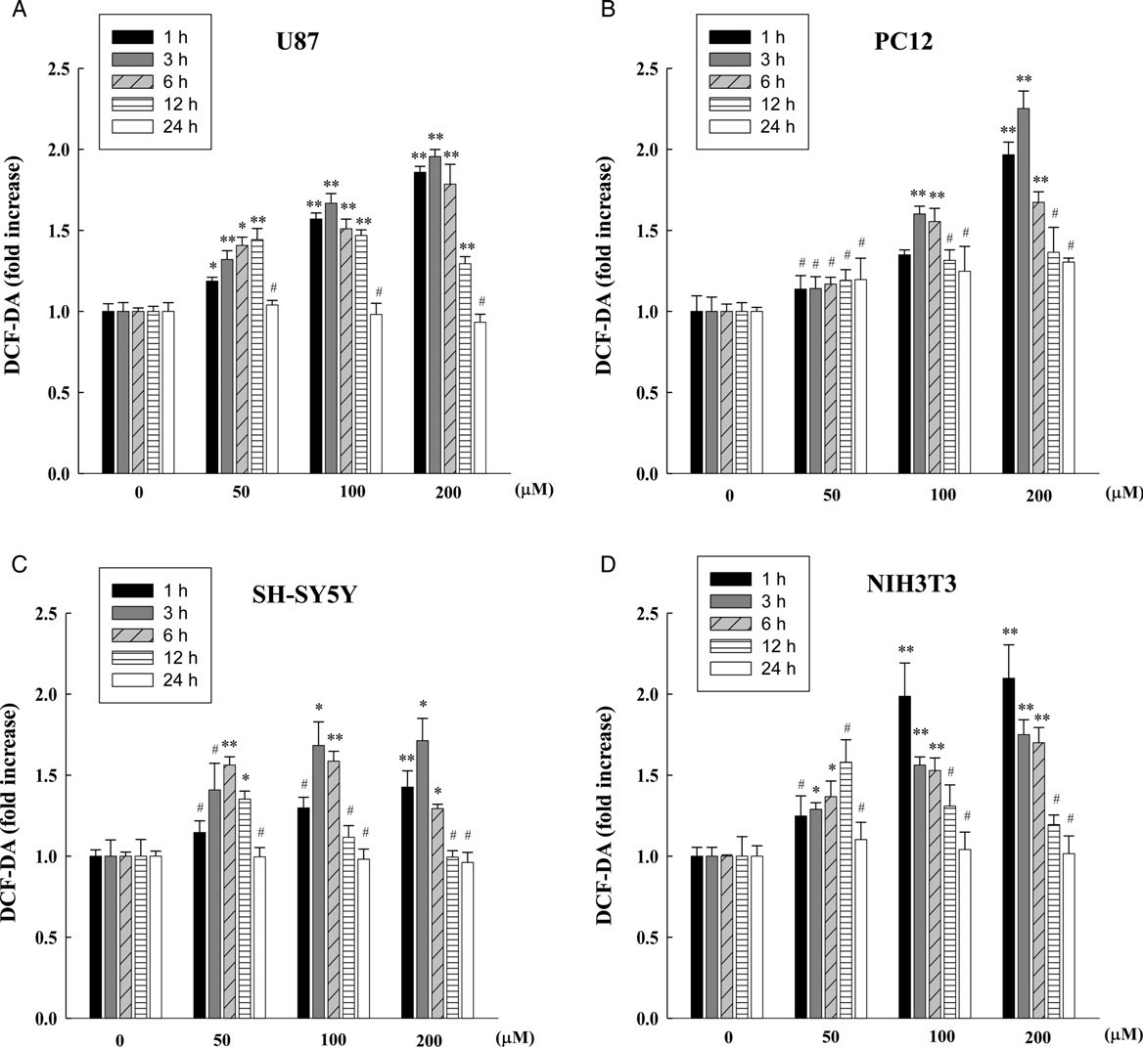
Measurement of ROS levels in H_2_O_2_-treated neuronal cells. ROS levels were measured by DCF-DA staining in 0, 50, 100 and 200 µM of H_2_O_2_-treated U87 (**A**), PC12 (**B**), and NIH3T3 cells (**D**), or in 0, 100, 200 and 400 µM of H_2_O_2_-treated SH-SY5Y cells (**C**) at indicated times after exposure. The data are expressed as the means of six independent experiments together with the standard deviations of the means (M ± SD). Statistical calculations were performed by ANOVA via the Tukey *post hoc* test. Statistical significance values were *P < 0.05 and **P < 0.01, compared with the untreated control at the same time-point. # indicates non-significance (*P* > 0.05). The presented significances were selected from multiple comparisons.

**Fig. 3.**
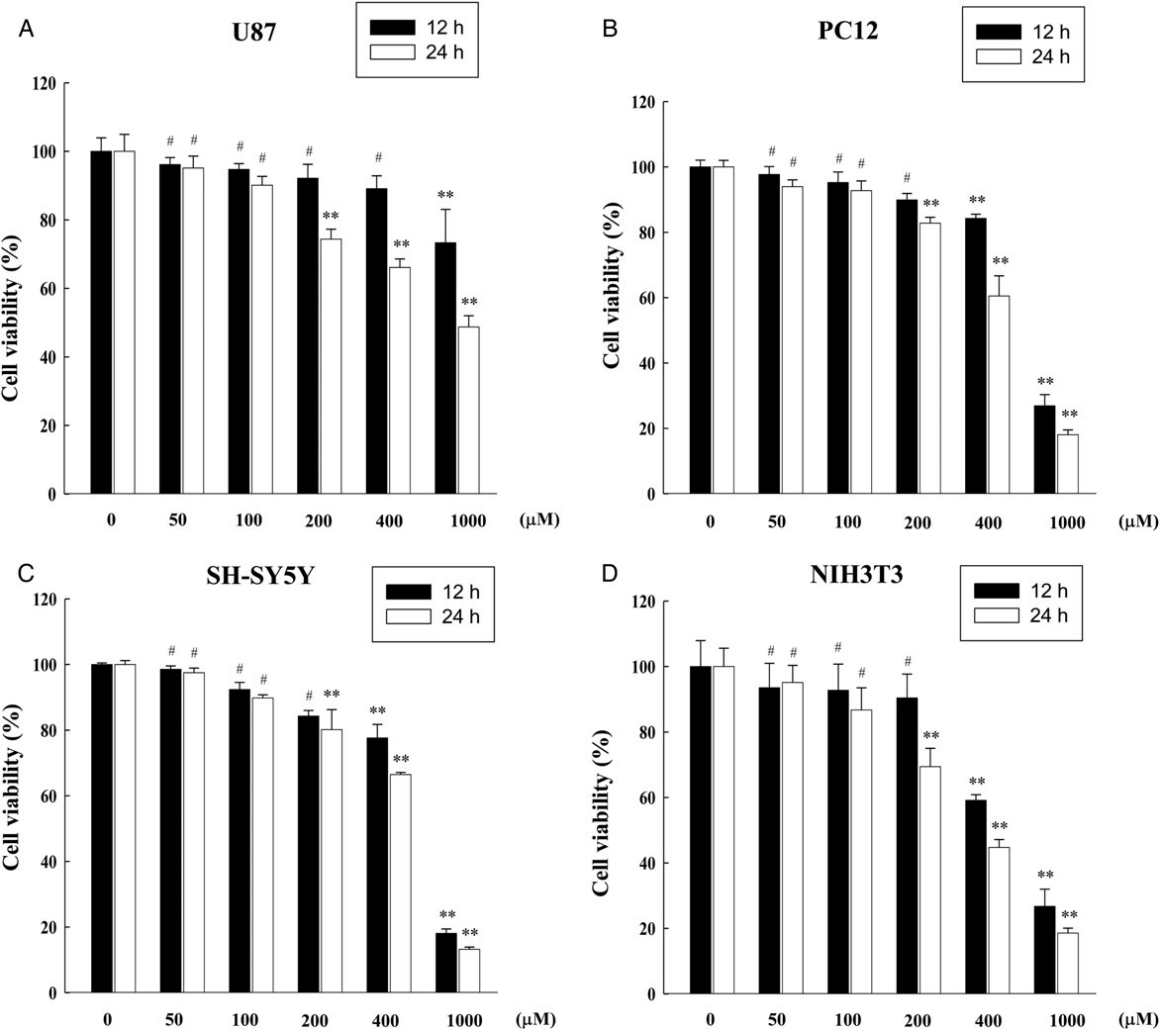
Cell viabilities of H_2_O_2_-treated neuronal cells. Cell viability was measured by MTT assay in U87 (**A**), PC12 (**B**), SH-SY5Y (**C**), and NIH3T3 cells (**D**) at 12 and 24 h after different doses of H_2_O_2_ treatment (0, 50, 100, 200, 400 and 1000 µM). The data are shown as the means of six independent experiments together with the standard deviations of the means (M ± SD). Statistical calculations were performed by ANOVA via the Tukey *post hoc* test. The statistical significance value was **P < 0.01, compared with the untreated control at the same time-point. # indicates non-significance (*P* > 0.05). The presented significances were selected from multiple comparisons.

To investigate the effect of co-exposure with combined RF radiation and the ROS inducer H_2_O_2_, we first wanted to determine the optimal H_2_O_2_ concentration for the co-exposure. ROS levels were determined by DCF-DA staining at 1, 3, 6, 12 and 24 h in U87, PC12 and NIH3T3 cells after treatment with 0, 50, 100 and 200 µM H_2_O_2_ (Fig. 2). ROS levels in H_2_O_2_-treated U87, PC12 and NIH3T3 cells were increased in time- and dose-dependent manners up to a certain level of H_2_O_2_ concentration (Fig. 2A, B and D). Since SH-SY5Y cells are more resistant to oxidative stress compared with the other three cell lines, we treated SH-SY5Y cells with a range of doses of H_2_O_2_ including 0, 100, 200 and 400 µM then measured intracellular ROS levels (Fig. 2C). After treatment with various concentrations of H_2_O_2_ (0, 50, 100, 200, 400 and 1000 µM), cell viability was also measured by MTT assay in U87, PC12, SH-SY5Y and NIH3T3 cells 12 and 24 h post exposure (Fig. 3). From the statistical analyses by ANOVA, treatment with 100 µM H_2_O_2_ resulted in a statistically significant increase in the ROS level >1.5-fold and, at the same time, maintained cell viability above 80% up to 24 h post exposure (without statistical significance). Therefore, we selected 100 µM H_2_O_2_ concentration for the co-exposure of H_2_O_2_ and combined RF radiation.

### Effect of combined RF and H_2_O_2_ co-exposure on ROS levels in neuronal cells

**Fig. 4.**
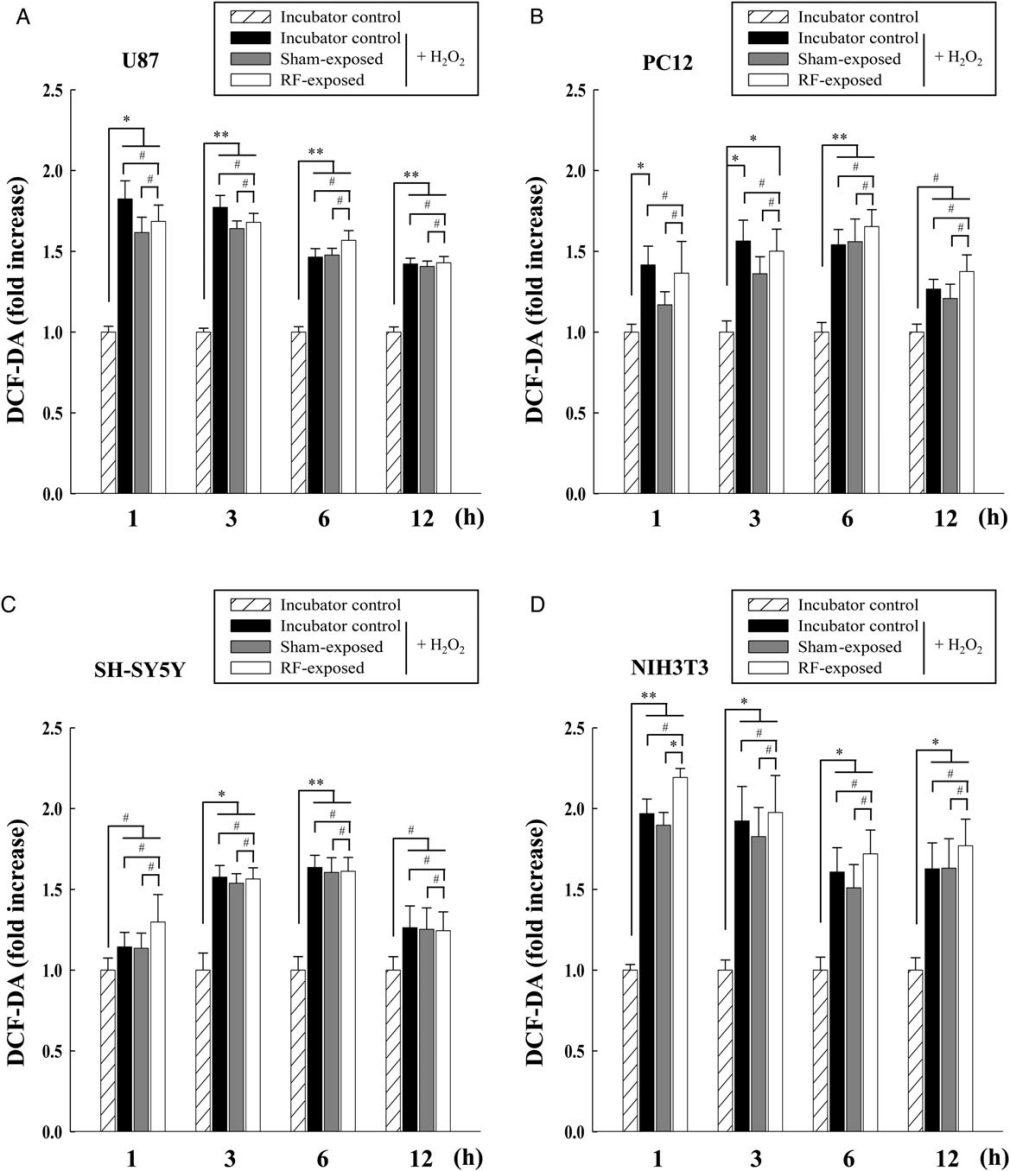
Measurement of ROS levels in neuronal cells after co-exposure to multiple RF signals (CDMA at 2 W/kg plus WCDMA at 2 W/kg for 2 h) and 100 µM H_2_O_2_. ROS were measured by DCF-DA staining in U87 (**A**), PC12 (**B**), SH-SY5Y (**C**), and NIH3T3 cells (**D**) at indicated periods after exposure. The data are shown as the means of six independent experiments together with the standard deviations of the means (M ± SD). Statistical calculations were performed by ANOVA via the Tukey *post hoc* test. Statistical significance values were *P < 0.05 and **P < 0.01, compared with either the incubator control group with or without H_2_O_2_, or the sham-exposed group with H_2_O_2_ treatment at each time-point. # indicates non-significance (*P* > 0.05).

We investigated the effects of co-exposure to combined RF radiation and H_2_O_2_ on ROS levels in three different neuronal cell lines (U87, PC12 and SH-SY5Y) and additionally in NIH3T3 mouse fibroblast cells. Cells were exposed to combined RF radiation (CDMA at SAR of 2 W/kg plus WCDMA at SAR of 2 W/kg) for 2 h prior to 100 µM H_2_O_2_ treatment, and then incubated for the indicated periods for the ROS measurements. ROS levels in cells exposed to combined RF radiation with H_2_O_2_ showed no statistically significant changes compared with incubator control or sham-exposed groups of 100 µM H_2_O_2_-treated cells (Fig. 4A–C). We could only detect a statistically significant change at 1 h co-exposure of RF radiation and 100 µM H_2_O_2_ in NIH3T3 cells (Fig. 4D). Conclusively, from our statistical analyses using ANOVA, we observed that combined RF exposure provided no significant additive effects on the ROS level changes induced by treatment with H_2_O_2_ in four different kinds of cells, with the exception of 1 h post co-exposure to RF radiation and H_2_O_2_ of NIH3T3 cells.

### Effect of menadione treatment on ROS level and cell viability in neuronal cells

**Fig. 5.**
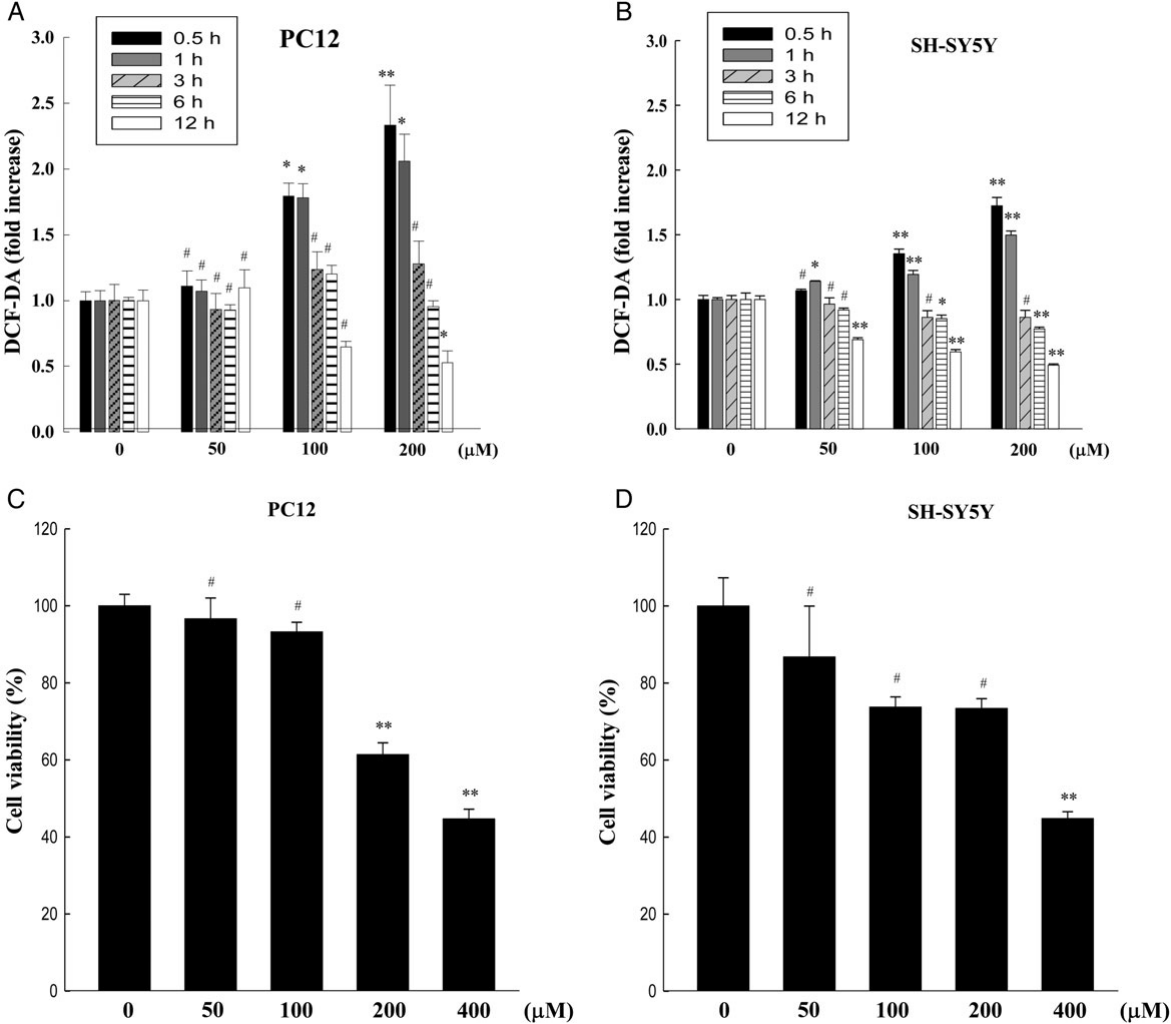
ROS levels and cell viabilities in menadione-treated neuronal cells. ROS levels were measured by DCF-DA staining in PC12 (**A**) and SH-SY5Y cells (**B**) after a range of concentrations of menadione treatment at indicated time-points. Cell viabilities were measured by MTT assay in PC12 (**C**) and SH-SY5Y cells (**D**) after different concentrations of menadione treatment for 24 h. The data are expressed as the means of six independent experiments together with the standard deviations of the means (M ± SD). Statistical calculations were performed by ANOVA via the Tukey *post hoc* test. Statistical significance values were *P < 0.05 and **P < 0.01, compared with untreated the control group at the same time-point. # indicates non-significance (*P* > 0.05). The presented significances were selected from multiple comparisons.

ROS levels and cell viability were assessed in four different cell lines (U87, PC12, SH-SY5Y and NIH3T3 cells) after menadione treatment. Since U87 and NIH3T3 cells did not show detectable changes in ROS levels up to 12 h after menadione treatment up to a concentration of 400 µM (data not shown), we measured the ROS level and cell viability only in PC12 and SH-SY5Y cells. In PC12 cells, from our statistical analyses using ANOVA, a statistically significant increase in the ROS level (>1.5-fold) was detected at 0.5 h and 1 h, but cell viability did not show a statistically significant change (still above 80%) at 24 h after 100 µM of menadione treatment (Fig. 5A and C). In SH-SY5Y cells, differences in cell viabilities did not reach statistically significant levels with 100 or 200 µM menadione treatments, but ROS levels were more evidently increased (>1.5-fold) with statistical significance after 200 µM menadione treatment than 100 µM treatment (Fig. 5B and D). Therefore, we selected 100 and 200 µM concentrations of menadione for the co-exposure experiments on PC12 and SH-SY5Y cells, respectively.

### Effects of combined RF radiation and menadione co-exposure on ROS levels in neuronal cells

**Fig. 6.**
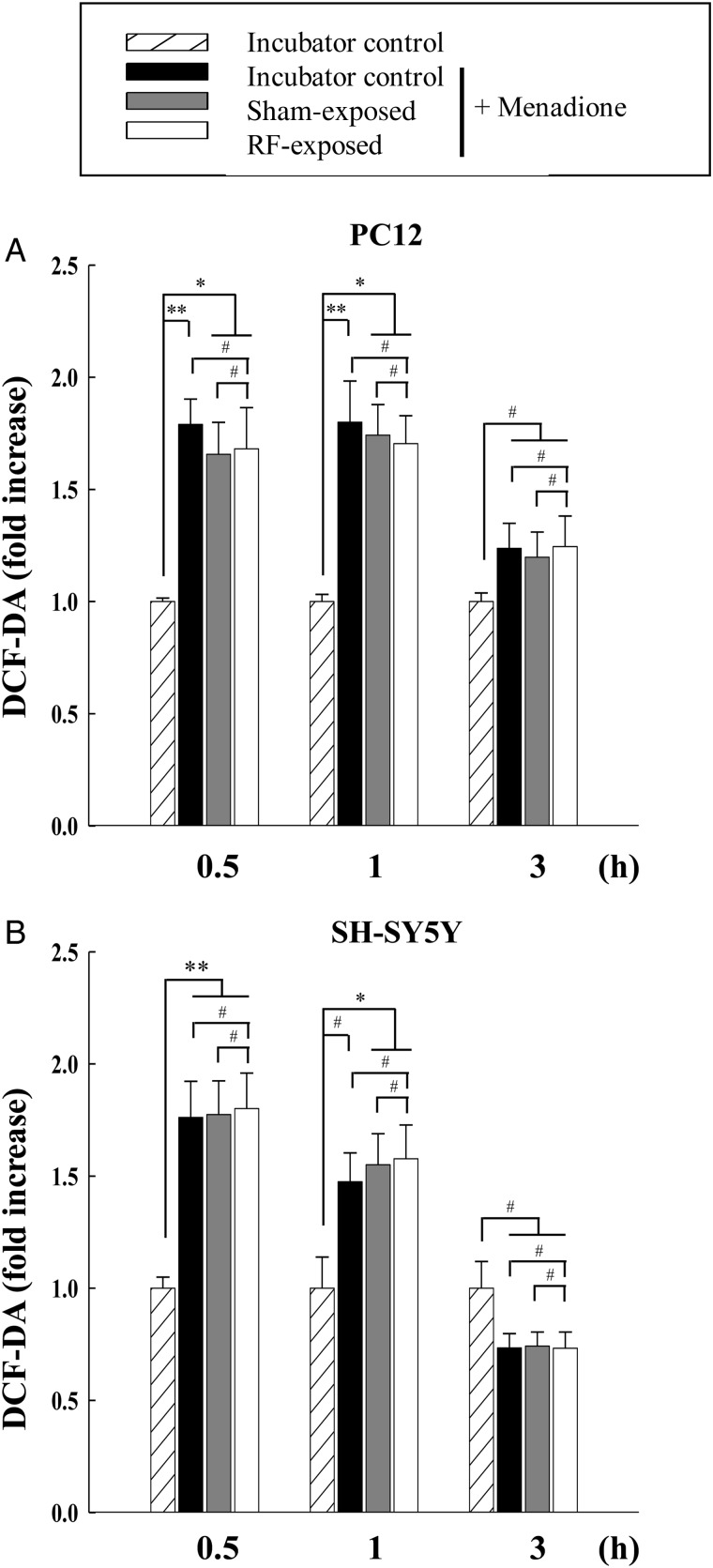
Measurement of intracellular ROS levels in PC12 and SH-SY5Y cells which were co-exposed to multiple RF signals (CDMA at 2 W/kg plus WCDMA at 2 W/kg for 2 h) and menadione. ROS were measured by DCF-DA staining in PC12 (**A**) and SH-SY5Y cells (**B**) at 0.5, 1 and 3 h after co-exposure. The data are expressed as the means of six independent experiments together with the standard deviations of the means (M ± SD). Statistical calculations were performed by ANOVA via the Tukey *post hoc* test. Statistical significance values were *P < 0.05 and **P < 0.01, compared with either the incubator control group with or without menadione, or the sham-exposed group with menadione treatment at each time-point. # indicates non-significance (*P* > 0.05).

To determine the effect of combined RF radiation and menadione co-exposure on ROS levels, we measured the ROS levels in PC12 and SH-SY5Y cells at 0.5, 1 and 3 h after treatment with 100 and 200 µM menadione in combination with combined RF radiation, respectively. From our statistical analyses using ANOVA, ROS were increased with statistical significance in incubator control and sham-exposed cells for 0.5 and 1 h after menadione treatment, however we could not detect any synergistic or additive effect when menadione was treated with combined RF radiation in both cell lines (Fig. 6A and B).

## DISCUSSION

Even though a considerable number of epidemiological, *in vivo* and *in vitro* studies have been conducted to provide scientific evidence for a health risk assessment of mobile phone radiation [22, 23], apart from our group's recent papers no reports regarding the biological effects of exposure to multiple RF radiation signals are available. We reported that combined exposure of CDMA and WCDMA RF radiation did not cause any observable adverse effects on mouse fetuses [24]. We demonstrated that combinations of CDMA signal at 837 MHz and WCDMA signal at 1950 MHz did not affect cell cycle progression *in vitro* [3]. We also observed no effect of combined RF radiation on ROS level, superoxide dismutase activity, or ratio of reduced/oxidized glutathione in human mammary epithelial MCF10A cells at 10, 24 or 48 h after exposure [25]. As an extension of our efforts to elucidate the biological effects of multiple RF radiation signals on oxidative stress, in this study we investigated whether co-exposure to multi-RF radiation signals and ROS inducers could modify ROS production in neuronal cell models.

The possibility that RF radiation might act as a co-stressor with well-known neurotoxic challenges, thus playing a role in neurodegenerative diseases, has so far been poorly explored. This could be potentially important, since no details are available on the possible role of RF radiation as an environmental factor affecting brain aging quality and the risk of neurodegenerative diseases. In particular, Del Vecchio and colleagues [16] tested the influence of RF radiation on glutamate toxicity, which is a final molecular mechanism in many neurodegenerative diseases including stroke, epilepsy, and perhaps Alzheimer's disease. They concluded that exposure to an RF signal acts as a co-stressor for the oxidative damage of neural cells but only under particular circumstances. They also found that RF radiation reduced the amount of neuritis generated by neuronal cells [26]. Hossman and Hermann [13] observed that most of the positive results reported so far could be attributed to thermal effects from the experimental conditions described in their review paper about the effects of RF radiation of mobile phones on the central nervous system.

Recently, oxidative stress has been suggested to be the underlying mechanism responsible for the reported cellular effects of RF radiation, because ROS affects a large number of physiological functions via damage of cell lipids, proteins, DNA and other intracellular macromolecules [27–30]. Oxidative stress-mediated molecular damage, as a result of excessive accumulation of ROS, contributes to the pathogenesis of several chronic diseases including cancer, atherosclerosis, stroke, rheumatoid arthritis, neurodegeneration and diabetes. DNA oxidation has been given more attention because DNA damage directly results in mutagenesis and carcinogenesis [31]. Oxidative damage to not only nuclear DNA but also mitochondrial DNA could contribute to human diseases. The most often measured index of oxidative DNA damage is 8-hydroxyguanine (8-OHG) or 8-hydroxy deoxyguanosine (8-OHdG) [32]. Some studies have demonstrated that mobile phone radiation induce ROS production or oxidative stress in human spermatozoa *in vitro*, in rat lymphocytes, in rat cornea and lens, and in human lens epithelial cells [33–36]. On the contrary, in other studies no significant difference in ROS production and no oxidative stress induction was observed after RF radiation exposures in fibrosarcoma cells, immune cells or spermatozoa [7–9, 11, 37, 38]. However, there are very few peer-reviewed scientific publications addressing RF radiation effects on ROS production, and there is a lack of consistent results in neuronal cells. Luukkonen *et al*. [18] exposed SH-SY5Y neuroblastoma cells to CW GSM 900 signals at 5 W/kg for 1 h (alone or in combination with menadione), which induced intracellular ROS production and DNA damage 30 and 60 min after the end of exposure. De Gannes *et al*. [30] indicated that exposure to EDGE signal RF radiation at 1800 MHz (2 and 10 W/kg for 1 and 24 h) did not induce ROS production in any of three human brain cell lines. The question as to the potential hazard posed by combined RF radiation has not yet been definitively answered for neuronal cells. In the light of these considerations, our study chose to investigate the ROS parameter as one of the most important cellular determinants known to affect a large number of physiological functions.

In this study, we present the results of a study aimed at assessing the effects of combined RF radiation (CDMA at 2 W/kg plus WCDMA at 2 W/kg for 2 h) on the intracellular ROS level in neuronal cell models: U87 human glioma cells, PC12 rat pheochromcytoma cells, and SH-SY5Y human neuroblastoma cells. Three experimental systems were used to test the hypothesis that multiple RF signals might act as promoters of ROS formation: combined (837 MHz and 1950 MHz) RF radiation alone, and combined RF radiation in combination with either H_2_O_2_ or menadione. H_2_O_2_ is a relatively stable ROS and is capable of diffusing through the cellular membrane [39]. H_2_O_2_ can generate the more detrimental hydroxyl radical (OH˙) or degrade into H_2_O and O_2_ via the enzymatic reaction of catalase and glutathione peroxidase [40, 41]. Thus, the combined action of these two enzymes provides a protective mechanism against oxidation of various intracellular components. Menadione (2-methyl-1,4-naphthoquinone: vitamin K3) has two major mechanisms for cytotoxic action in a variety of biological systems. First, menadione undergoes one-electron reduction by microsomal NADPH-cytochrome P-450 reductase and mitochondrial NADH ubiquinone oxidoreductase, yielding the corresponding semiquinone radicals. Under aerobic conditions, the semiquinone radicals participate in redox cycling to generate ROS such as the superoxide anion (O_2_^−^) and H_2_O_2_. Second, menadione is capable of reacting with the thiol groups of proteins and glutathione (GSH) [42–44]. In this study, we tested whether exposure to ROS inducers such as H_2_O_2_ and menadione as well as to combined RF produces additive or synergistic effects on ROS production. We observed that none of the responses to either combined RF alone or combined RF with ROS inducers showed additive or synergistic effects on ROS production. In our experiments, only a few conditions presented a slight increase in ROS in comparison with sham exposure, and those slight increases were not consistent or sustained. Living organisms possess natural defense systems of complex antioxidant mechanisms to detoxify intracellular ROS [45]. ROS is controlled through the activity of intracellular antioxidant enzymes such as superoxide dismutase (SOD), glutathione peroxidase (GSH-Px), glutathione reductase, and catalase (CAT) [46, 47]. Non-enzymatic antioxidants including carotenoids, flavonoids and related polyphenols, vitamins, and glutathione are also capable of neutralizing ROS and their actions. Antioxidants act at different stages and by different mechanisms for balancing of the intracellular ROS level [48]. Since various antioxidant defense mechanisms could help to maintain homeostasis in the face of a sudden burst of ROS production, we measured ROS accumulation at 0.5, 1, 3, 6 and 12 h after the RF radiation exposures, instead of measuring acute ROS generation. Although we currently cannot fully explain our results, they might reflect differences in the cell culture systems used, or could suggest that exposure to RF signals acts as a co-stressor for oxidative damage of neural cells only under particular circumstances, as suggested by Del Vecchio *et al*. [16].

## CONCLUSION

In conclusion, we found a transient small increase in the ROS level after exposure of neuronal cells to multiple RF signals (Fig. 1). However, we observed no prolonged or further increase in the ROS level. In addition, we observed no evident synergistic or additive effect of multiple RF signals on ROS generation when combined with additional treatment with ROS inducers. This was consistent with our previous report which demonstrated that single or combined RF radiation exposure alone did not elicit oxidative stress in MCF10A cells [25].

## FUNDING

This study was supported by the Korea Communications Commission (2012) grant funded by the Korean government.
